# Searching the best equilibrium moisture equation for lettuce seeds using measures of curvature and bias

**DOI:** 10.1002/fsn3.67

**Published:** 2013-10-02

**Authors:** Carolina T Bortolotti, Marcos A S Barrozo

**Affiliations:** Chemical Engineering School, Federal University of UberlândiaUberlândia, Minas Gerais, Brazil

**Keywords:** Equilibrium sorption, *Lactuca sativa*, nonlinearity measures

## Abstract

In the present work, we performed a discrimination analysis of the nonlinear equations used to describe sorption isotherms of the lettuce seeds. The equilibrium data were obtained by the static method using saturated salt solutions. The best model to describe the equilibrium moisture of lettuce seeds was selected using measures of curvature and bias. The obtained results showed that the Copace equation was the best model in terms of nonsignificance for bias and nonlinearity measures.

## Introduction

Seed drying is the first and most important stage in the processing of seed conservation. Low seed moisture content is very important for long-term storage and for seed longevity (Felipe and Barrozo 2003). Seeds lose germination and vigor in the storage mainly because of high moisture content (Barrozo et al. 2006). High moisture content increases the respiration rate of seeds. Respiring seeds may generate enough heat to kill the seeds quickly (Duarte et al. 2004).

The equilibrium moisture of a material in a specified temperature and relative humidity (RH) is given by the sorption isotherms. Sorption isotherms are important because they can be used to predict potential changes in biological materials stability (Barrozo et al. 2001).

To obtain experimentally the sorption isotherms by the static method, we should use acid solutions of different concentrations or saturated salt solutions. The salt solutions are used very often because of the facility to maintain the constant RH (Arnosti et al. 1999).

Most of the equilibrium moisture equations presented in the literature are nonlinear (Barrozo et al. 1996; Tolaba et al. 2004; Pagano and Mascheroni 2005; Ribeiro et al. 2005; Barrozo et al. 2006; Cordeiro et al. 2006; Arruda et al. 2009). Thus, in this case, care should be taken when estimating their parameters, as in some situations, the estimators could not be appropriate. In this way, there are in the literature some procedures to validate the statistical properties of the least squares (LS) estimators of nonlinear models.

The main objective of the present work is to use the nonlinearity measures, to select, from six model equations, the best equation to represent the sorption equilibrium isotherms of lettuce seeds.

## Sorption Equilibrium Equations

A large number of theoretical equations, empirical or semi-empirical, has been proposed in the literature to estimate the equilibrium moisture content, *M*_eq_, of agricultural and food products as a function of the air RH, and the solid material temperature, *T*_*s*_. The theoretical equations are based on well-known sorption kinetics theories, for example, the equations of Kelvin, Langmuir, and BET. In many cases, the theoretical models cannot predict with accuracy the equilibrium moisture content of seeds over a wide range of temperature and air RH. This fact has led some authors to use empirical or semi-empirical equations to try to increase the accuracy of the predicted values of *M*_eq_. The most important equations introduced in the literature to predict the equilibrium moisture of biological materials are given in Table 1. The *a*, *b*, *c*, and *d* coefficients are the model parameters to be fitted to experimental data. These models have been tested and their parameters evaluated for many agricultural crops (Barrozo et al. 1996; Arnosti et al. 1999; Tolaba et al. 2004; Pagano and Mascheroni 2005; Ribeiro et al. 2005; Cordeiro et al. 2006). Some of these equations, among others, have been adopted as standard equations by the American Society of Agricultural Engineers for describing sorption isotherms (ASAE 2007).

**Table 1:** The six most frequently cited equations for predicting the equilibrium moisture content

Name	Equation	Reference
Henderson	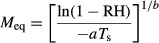 (1)	Henderson (1952)
Henderson–Thompson	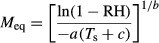 (2)	Thompson et al. (1968)
Chung–Pfost	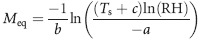 (3)	Chung and Pfost (1967)
Chen–Clayton	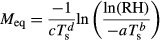 (4)	Chen and Clayton (1971)
Sabbah	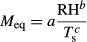 (5)	Pfost et al. (1976)
Copace	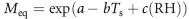 (6)	Corrêa et al. (2007)

Mazza and Jayas (1991) used the Chung–Pfost equation (eq. 3) to describe the equilibrium moisture content of pea seeds. Chen (2003) showed that the Henderson–Thompson equation (eq. 2) was the best model to represent the experimental data of sorption of pea. Hutchinson and Otten (1983) obtained good fit of the Henderson and Chung–Pfost equations when describing the equilibrium moisture of the white beans. Araujo et al. (2001) studying the desorption of sweet corn seeds obtained the best results with the Sabbah and Chung–Pfost equations.

The choice of the best models in these previous works was generally based on *R*^2^ values and residual analysis. However, these criteria may be insufficient to discriminate between nonlinear regression equations like equilibrium sorption isotherms models (Conceição Filho et al. 1998). Other properties are desirable for nonlinear models, such as the LS parameters should be almost unbiased, normally distributed, and its variances should be close to the minimum variance (Lira et al. 2010). In the present work, the statistical discrimination among the sorption equilibrium equations is based on nonlinearity measures.

## Materials and Methods

### Material

The lettuce seed (*Lactuca sativa* L.) is an achene, that is, a dry, indehiscent (the fruit does not open on its own to release seeds), one seeded fruit, that consists of an elongated dicotyledonous embryo surrounded, from inside to out, by the three successive layers: endosperm, integument (seed coat), and pericarp (fruit wall).

### Experimental methodology

The technique used to obtain the equilibrium data of lettuce seed was the static method with saturated salt solutions. Some salts were chosen, to get a wide range of RH (Greenspan 1977).

Approximately 1 g of lettuce seeds were sealed up in small cylindrical glasses containing different salt solutions, corresponding to a range of RH of 11–95% (Table 2). The containers were 60 mm height and 40 mm wide, with a perforated tray 30 mm over the bottom of the container to sustain the seeds and avoid contact with the solution. This amount of seeds was used to obtain a monolayer on the tray.

**Table 2:** Relative humidity for the salt solutions as function of temperature

	Temperature (°C)			
	
Salt solution	30	40	50	60
Lithium chloride	0.113	0.112	0.111	0.110
Potassium acetate	0.216	0.204	0.192	0.160
Magnesium chloride	0.324	0.318	0.312	0.306
Potassium carbonate	0.432	0.432	0.433	0.432
Sodium nitrite	0.635	0.616	0.597	0.578
Sodium chloride	0.750	0.748	0.746	0.745
Potassium chloride	0.950	0.950	0.950	0.950

In order to guarantee the desorption, the moisture of the lettuce seed was always higher than the equilibrium value. The containers were placed in an oven at temperatures of 30, 40, 50, and 60°C with a maximum variation of 0.5°C, kept under constant thermodynamic conditions. Measurements of the mass sample were taken every 48 h and these samples were weighed on an analytical balance with precision of 1 mg. The equilibrium moisture of each sample was found by the oven drying method at 105 ± 3°C for 24 h. When three subsequent measurements resulted in identical values, the equilibrium conditions were found. To ensure the reliability of the results, each salt solution was used three times in every condition (Barrozo et al. 1996).

### Statistical methodology

#### Nonlinearity measures

A question that is very important is how well a specified model fits the data. Some techniques have been studied in the literature to investigate whether a specified equation provides a good description of the experimental data and if there is statistical reliability for the estimated parameters.

Nonlinear regression models differs in general from linear regression models in that the LS estimators of the parameters are biased, nonnormally distributed, and have variances exceeding the minimum possible variances. The extent of the bias, nonnormality, and excess variance differs widely from model to model. When the LS estimators of nonlinear models present small bias, near normal distribution, and almost constant variances, it can be stated that the estimators present a near linear behavior and, consequently, the inferences will be more reliable (Arnosti et al. 1999). The extent of nonlinear behavior can be evaluated through nonlinearity measures.

Nonlinearity measurements are used in the literature to evaluate adequability of the linear approximation and their effects on the inferences (Seber and Wild 1989). One of the first relevant attempts to quantify the nonlinearity of a nonlinear regression was presented by Beale (1960), who proposed four measures. However, these measures should not be used in practice, as they tend to underestimate the true nonlinearity (Seber and Wild 1989). Box (1971) presented a formula for estimating the bias in the LS estimators. Bates and Watts (1980) proposed some measures of nonlinearity based on the geometric concept of curvature. They separated the nonlinearity of a model in two components: one associates with the curvature of the solution locus, called “intrinsic” nonlinearity (IN) and another associated with the fact that the projections of the parameters lines on the tangent plane to the solution locus are neither straight, parallel, nor equispaced, called “parameters effect” (PE). Bates and Watts (1980) also showed that the bias proposed by Box (1971) is closely related to their measure of PE curvature.

#### Curvature measures of nonlinearity proposed by Bates and Watts (1980)

Intrinsic nonlinearity (IN), which is characteristic of the model, measures the curvature of all solutions in the sampled space. A linear regression model presents a nil (IN) measure, as the estimation space is a straight line, a plane, or a hyperplane. In contrast, the estimation space of nonlinear model is curved, and (IN) measures the “maximum intrinsic curvature.” A negligible IN will mean a negligible bias in the predicted values of response.

Nonlinearity due to PE depends on the sequence that the parameters appear in the model. The measure (PE) measures the unequal spacing and lack of parallelism of parameter lines projected on to the tangent plane to the solution. In the linear case, the parameter lines are parallel. When the nonlinearity is mainly due to the PE, a reparameterization becomes useful. If the PE nonlinearity is negligible, the statistical tests of the consistency of the parameters will be valid.

The statistical significance of (IN) and (PE) may be assessed by comparing these values with 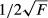, where *F* = *F* (*p*,*n* − *p*,α) is obtained from a table of the F-distribution corresponding to significance level. The solution locus may be considered to be linear within an ∼95% confidence region if IN 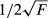 (α = 0,05). In the same way, if PE 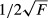, the parameter lines projected on to the tangent plane to the solution may be considered as being sufficiently parallel and uniformly spaced, hence the tests of parameter invariance will be adequate.

A computer program in Fortran language was used to perform all the calculations required to determine the (IN) and (PE) values.

#### The bias measure proposed by Box (1971)

Box (1971) proposed the following equation for calculating the bias in the LS estimates of the parameters in nonlinear models:


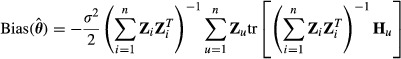
(7)

where 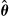 represents the LS estimator, **Z**_*i*_ and **Z**_*u*_ is the *p* × 1 vector of first derivatives of *f* (**X**_*i*_, **)** and **H**_*u*_ is the *p* × *p* matrix of second derivatives with respect to each of the parameters, evaluated at **X**_*i*_ (independent variable, which in this case are RH and *T*_s_), where *i* = 1, 2, …, *n*, and *n* is the data number.

The bias expressed as a percentage of the LS estimate is a useful quantity, where an absolute value in excess of 1% indicates nonlinearity behavior. Thus, this nonlinearity measurement can be useful for identification of which parameter, or parameters, are responsible for the nonlinearity behavior. The percentage bias is given by


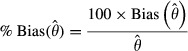
(8)

## Results and Discussion

Table 3 shows the results of LS estimation (for *M*_eq_ in dry base, *T* in °C and RH decimal fraction), with the respective values for *R*^2^ and the ratio *F*, as the intrinsic curvature measure (IN), the PE measure, and the bias of Box, for all considered models.

**Table 3:** Statistical results of the least squares estimation

Equation	*R*^2^	Curvature	Parameter	Estimated value	% Bias box
Henderson (1)*	0.882	IN = 0.038	a	9.76 × 10^−4^	1.39
		PE = 1.64	b	1.775	0.25
Henderson–Thompson (2)**	0.956	IN = 0.032	a	1.03 × 10^−4^	0.21
		PE = 191.2	b	1.47	0.06
			c	528.58	91.99
Chung–Pfost (3)**	0.968	IN = 0.024	a	921,6	32.73
		PE = 2.46	b	0.29	0.06
			c	232.23	37.56
Chen–Clayton (4)***	0.969	IN = 0.090	a	8.43	17.38
		PE = 23.94	b	−0.25	0.49
			c	0.37	5.36
			d	−0.068	0.63
Sabbah**	0.936	IN = 0.041	a	22.138	2.4505
		PE = 5.625	b	0.8741	0.0618
			c	0.1107	0.001
Copace**	0.954	IN = 0.026	a	1.2302	0.0368
		PE = 0.044	b	0.0035	0.0560
			c	1.722	0.0225

^*^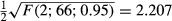; ^*^^*^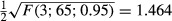; ^*^^*^^*^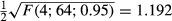, where *F* is the inverse of the probability Fisher distribution.

A higher *R*^2^ value for the Chen–Clayton model is not sufficient to assure the statistical validity of the LS estimators. It can be observed in Table 3 that all intrinsic curvature measures (IN), when compared to the value 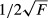, lf are not significant for the considered nonlinear models, which indicate small nonlinearity for the “solution locus” (Ratkowsky 1983). It is also observed in these results that the PE curvature measures are significative for equations (1–5) (PE 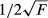), showing that at least one parameter for these equations, has a strong nonlinear behavior.

As equations (1–5) show significant nonlinear PE, the bias measures could show which parameters are responsible for this behavior (% bias >1%). The largest bias is given by equation (2) (Henderson–Thompson). It also can be observed in Table 3 results, that the inclusion of a third parameter (*c*) in the Henderson equation, that originated the Henderson–Thompson equation, implied in a larger nonlinear behavior. As this parameter is empirical, a reparameterization could be considered to solve this problem. The first five equations presented at least one parameter with a percentage bias higher than 1%, as the (PE) results have predicted. Therefore, for the sorption data of lettuce seeds, only the Copace equation presented a negligible bias for all fitted parameters. Thus, only for this equation it is possible to assume the valid inference results based on asymptotical approximations for the LS estimators.

**Figure 1:**
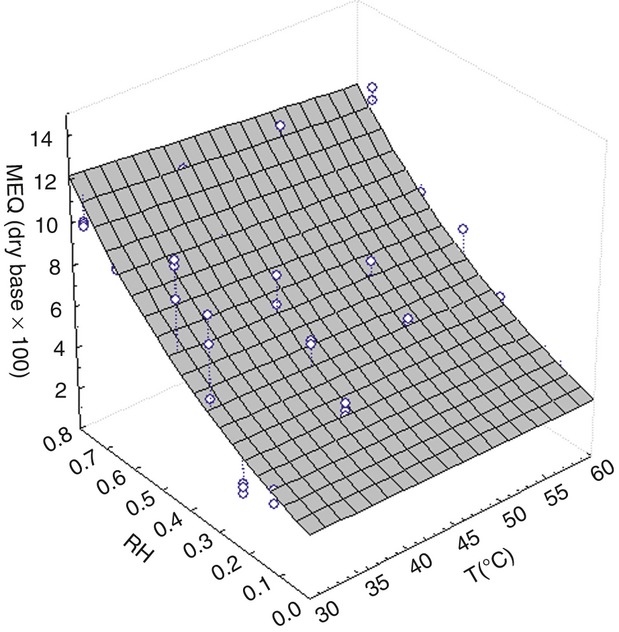
Experimental equilibrium moisture content of lettuce seeds as a function of RH and *T*_s_ and the response surface predicted by the Copace modified equation (eq. 6 in Table 1). RH, relative humidity; *T*_s_, solid material temperature.

In Figure 1, we have the response surface given by the Copace equation and the experimental equilibrium observations. We observe a good agreement between the data and the predicted values.

## Conclusions

In this work a discrimination approach based on nonlinearity measures was used to get the best equation to represent equilibrium data of lettuce seeds; this methodology could be extended to other nonlinear models.

The Copace equation is the only one that presented curvature measures and bias not significant. Therefore, this equation is the most appropriate to represent equilibrium data for lettuce seeds.
